# The public impact of academic and print media portrayals of TMS: shining a spotlight on discrepancies in the literature

**DOI:** 10.1186/s12910-022-00760-5

**Published:** 2022-03-13

**Authors:** Abigail Scheper, Cynthia Rosenfeld, Veljko Dubljević

**Affiliations:** 1 North Carolina State University, 101 Lampe Drive (Withers Hall 453), Raleigh, NC 27695 USA; 2 University of Virginia School of Law, Charlottesville, USA

**Keywords:** Transcranial magnetic stimulation (TMS), Media, Ethics, Agenda setting

## Abstract

**Background:**

Transcranial magnetic stimulation (TMS) is an FDA approved treatment for major depression, migraine, obsessive compulsive disorder, and smoking addiction. TMS has gained popular media support, but media coverage and commercial reporting of TMS services may be contributing to the landscape of ethical issues.

**Methods:**

We explore the differences between the academic and print media literature portrayals of TMS to evaluate their ethical impact for the public. We performed a comprehensive literature review using PubMed and NexisUni databases to evaluate the literature available on TMS from 2014 to 2019. Our sample consisted of 1632 academic articles and 468 print media articles for a total of 2100 articles. We then coded each article for seven specific top-level codes: (1) type of source, (2) year of publication, (3) purpose of TMS application, (4) age of subjects, (5) population, (6) overall tone, and (7) specification of TMS parameters. We also made some additional notes of the TMS parameters where specified and the breakdown of mental health applications.

**Results:**

Our results indicated several discrepancies between the academic and the print media reporting about TMS technology, particularly with regards to tone and specificity. Namely, the academic sample was largely neutral and specific about the parameters under which TMS was being applied, while the print media sample was heavily optimistic and presented the application of TMS with far less specificity. There was some convergence between the two samples, such as the focus of both on therapy as the predominant TMS application.

**Conclusions:**

We call upon the academic community to increase scrutiny of TMS services in order to ensure that people’s knowledge of health technologies is not unduly influenced by sensational claims and a general lack of adequate information.

## Background

In recent years, rapid development of new noninvasive neurostimulation techniques has generated claims of therapeutic and enhancement potential that has captivated public attention [1]. Among them is transcranial magnetic stimulation (TMS), a form of neurostimulation [2] that uses magnetic fields to influence brain activity in a myriad of applications [3]. TMS is approved by the Food and Drug Administration (FDA) as a treatment for drug-refractory major depression (MD), migraine headaches, obsessive compulsive disorder (OCD; [4]), and most recently nicotine addiction [5]. However, recent surveys of the literature reported that TMS is being used to treat various conditions outside of these approved applications. These include a range of neuropsychiatric (e.g., bipolar, generalized anxiety, and post-traumatic stress disorder (PTSD), see [6]), volitional (e.g., various forms of substance misuse, see [7]), eating (see [8]), and neurodevelopmental or neurodegenerative disorders (see [9]).

For more than a decade, researchers have noted the increasing popularity of TMS as a treatment option [10, 11] and as an area of clinical and academic study with highly variable results [12]. During this time of increased therapeutic and academic popularity, there have been accompanying direct-to-consumer appeals surrounding TMS treatment, such as marketing and advertising firms who claim to generate 30 or more new TMS calls a month for clinicians using their services [13]. This therapeutic landscape provokes the motivating concern for this paper, namely *how are media outlets contributing to the messaging surrounding TMS treatments?* More specifically, our concerns about media messaging are twofold: (1) how and to what extent do media stories accurately represent the available evidence for the efficacy, applicability, and potential side effects of TMS, and (2) how and to what extent does the tone of media coverage contribute to information dissemination or promotion of TMS treatments to the public?

The concerns about media messaging stems from research on (1) the landscape of ethical issues surrounding TMS and (2) the influence of media on public perception. First, there are the ethical issues surrounding TMS. The safety of TMS in controlled research settings has been reported in a number of research studies. Guidelines for safety in experiments using TMS were developed in 1998 [14] and updated in 2009 [15] and again in 2020 [16]. The Clinical TMS Society has recently developed guidelines for safe application of TMS in clinical settings for the treatment of Major Depressive Disorder [17].^1^ Recently, Kim and colleagues [18] published a literature review on the use of TMS for treatment of psychiatric disorders and for enhancement purposes. They found the optimal parameters for applying TMS have yet to be determined. Further, they concluded that while TMS is a promising technology, more research is needed on the long-term effects of treatment, as well as addressing the discrepancies in research on TMS’s impact on cognitive functioning. Whether some TMS application is appropriate depends on the scientific community reaching a consensus about the paradigms that create effective results.

A recent report by Wexler and colleagues [19] analyzed promotion of TMS on provider websites. While device manufacturers cannot legally promote TMS for off-label indications, providers don’t have such legal restrictions. Noting that their findings are underestimating this phenomenon, Wexler and colleagues report that over a hundred clinics in the U.S. currently promote TMS for non-indicated uses, notably for anxiety (67.3%), PTSD (59.6%), ADHD (23.1%) and cognitive or performance enhancement (8.7%). When discussing ethical aspects of TMS therapy, it is important to note that TMS is associated with certain risks in addition to the benefits it provides. TMS has several harmful potential side-effects, including induced seizures, syncope, transient induction of hypomania, discomfort or pain, cognitive changes, hearing loss, and transient impairment of working memory [20]. Adverse effects resulting from long-term, repeated exposure to TMS are similarly important to note. These effects include not only unknown complications due to TMS-induced fields but also other effects, such as potential hearing impairments due to the noise levels produced by TMS coils that can exceed 140 dB [15]. While the harm/benefit ratio may be favorable in some cases of TMS therapy (e.g., where the risk of severe side effects is low while the potential for benefit is high, such as in cases of drug refractory depression), it is imperative to explore how patients are actually informed about side effects, particularly more severe ones (e.g., seizures). While these side effects are known issues to researchers of TMS, it is unclear whether the general public is receiving this critical information, which makes an inquiry into the publicly-available information regarding TMS important and timely. A search of the information on TMS therapy on the internet provides a glimpse into contrasting “hype and hope” and “gloom and doom” perspectives ([21], at p. 69) that have such a distorting potential. At this interaction point, the existence of multiple novel forms of TMS blurs the line between research and therapy and can increase risks due to TMS-drug interactions, comorbidities, and unknowns about prevalence of (serious) adverse effects and side effects as elucidated by the FDA [19].

Second, there are concerns about media coverage and commercial reporting of TMS services contributing to the landscape of ethical issues through media’s ability to inform and influence public perception. Agenda-setting theory (AST; [22]) describes how media impacts what the public considers to be a major issue. Bernard Cohen [23] offered what is considered to be the classic summary of agenda setting: “The press may not be successful much of the time in telling people what to think, but it is stunningly successful in telling its readers what to think about” ([23]; at p. 13). Issues that are most salient—defined by frequency, location, and/or length of coverage—in the media become part of the public discourse [24]. The more coverage an issue receives, the more the public pays attention [25]. Media are further found to impact the public’s awareness of an issue based on *how* they report on a subject [22], including matters of health communication. Guo and Vu [26] conducted a longitudinal analysis on new indices and public opinion polls from 2001 to 2010 and found that news media, especially print media, had some agenda-setting effects on the public’s health priorities.

It is important to assess whether media coverage of TMS adequately reflects the sparse technical and regulatory guidelines for using this technique (e.g., [27] guideline is from [17]) and to consider what associated social (e.g., potential pressure from familial and other intimate relations to try this treatment they saw advertised) and ethical (e.g., concerns over inducing consumers to a treatment that may be unaffordable and unrecommended) issues might arise from direct-to-consumer messages about TMS treatments. While TMS could be therapeutically legitimate for a number of people with different health concerns, access to care may be influenced by misleading media portrayals and direct-to-consumer communications that offer overly positive or overly negative perspectives. Positively-biased information may lead to unrealistic expectations which fail to materialize for the majority of patients, ultimately leading patients to see their results as a “let-down.” For example, media that paints TMS as “life-changing” or a “miracle cure” for depression may unwittingly set up people for disappointment, given that a significant portion of people treated with TMS receive only mild benefit [20]. Similarly, as in situations of direct-to-consumer advertising (cf. [27, 28]), such stories could encourage patients to seek out treatment that is inappropriate for them. Negative perspectives, on the other hand, may discourage patients from seeking or continuing treatment, even if the technology has legitimate potential benefits. Given the implications that media representation can have for potential TMS patients, it is important that media take a balanced approach to discussing health technologies like TMS.

Given this previous research, we have chosen to compare print media and academic articles on TMS to get a better understanding of the potential ethical concerns at issue in media reports of TMS treatments. By exploring the case example of TMS, this study offers an in-depth look at the ethical implications of agenda-setting theory for health issues, particularly those issues that present themselves when discrepancies between print media and academic articles occur. In this paper, we sought to comprehensively track the relevant differences between the academic and print media literature portrayals of TMS in order to evaluate the ethical issues that arise from any discrepancies. Our work builds on previous reviews of TMS done by Luber and Lisanby [3] and Kim and colleagues [18]; our prior work on media representation of transcranial direct current stimulation (tDCS), another form of non-invasive brain stimulation [1]; and on research on health communication and agenda-setting theory [26]. Since the TMS literature predominantly covers the technology in earlier years, we tracked the media coverage of TMS during five years of uninterrupted research and clinical practice (from 2014 to 2019) in both the academic and print media literature to examine the most relevant information available for TMS. Additionally, unlike previous work, we included TMS used for all purposes, people, and conditions, as opposed to focusing on a single application or group (e.g., enhancement). Finally, our inclusion of the print media in our search sets this article apart from other reviews of TMS and focuses on our research question: What ethical issues arise when there are discrepancies in the factual information presented between print media and academic literature?

## Methods

We used qualitative methods to study the ethics of the presentation of TMS in the literature. In order to assess the publicly available information regarding TMS, we performed an extensive database search that allowed us to analyze the full body of relevant texts that have been published from 2014 to 2019 in both the academic and print media literature. Focusing first on the academic literature, we used the PubMed database to search for “Transcranial Magnetic Stimulation” and the relevant MESH terms^2^ in the period from January 1, 2014 to December 31, 2019. From this, we yielded 2273 articles for our retrieved sample. We then applied the following exclusion criteria to the retrieved sample: (1) papers without an English abstract or available full text in English; (2) papers detailing techniques other than TMS; (3) papers reporting the use of TMS during surgery or on patients under anesthesia; (4) TMS applied to nonhuman models (e.g., rat models); (5) TMS applied to areas other than the brain/outside of typical TMS scope; and (6) unpublished proofs. These criteria led to the exclusion of 641 articles, leaving a final count of 1632 relevant papers.

Applying similar techniques, we used the NexisUni database to gather a comparable print media sample. Print media sources were selected exclusively for three primary reasons. First, focusing on print media allowed us to create a manageable dataset for analysis. For a simple comparison, as of 2018, there are approximately 1300 daily print newspapers in the United States [28] compared to the ever-increasing volume of news sources online, which range from the online platform of *The New York Times* to social media accounts [29]. Second, print media remains more conducive to systematic study owing to the refined filtering capacities of databases at university libraries and platforms like NexisUni. Third, print media remains better preserved in accessible archives. A recent study showed that a quarter of links of a major media outlet like *The New York Times* were corrupted—meaning the links were dead and the linked pages were either deleted, changed, or moved without HTML redirection [30]. Focusing on print media afforded the opportunity to create a more stable and more replicable archive, which will benefit future studies.

We used the search terms “‘transcranial magnetic stimulation’ and (enhancement or therapy)” from the period of January 1, 2014 to December 31, 2019, and limited our search to the categories of “newswires & press releases,” “newspapers,” and “magazines & journals” in order to gather the full body of print media articles. This search yielded 1420 print media articles. Here, our exclusion criteria were as follows: (1) sources targeting medical professionals; (2) irrelevant article types (e.g., obituaries, clinical trial reports, economic/market reports); (3) articles primarily detailing another neurostimulation/treatment technique; (4) duplicates; (5) online-only articles; and (6) articles unavailable in English. After applying these criteria, the final print media sample was 468 relevant papers (952 excluded). Our final sample thus consisted of 2100 articles in total, which we kept separated into an academic sample and a print media sample.

We organized both the academic and print media samples by publication date, sorting them from oldest to most recent, and selected every tenth article from these two compilations to create two pilot samples. Doing so provided us with a sample that was 10% of the size of the overall body of data (163 academic articles and 47 print media articles), making a more manageable sample for analysis that would allow us to establish inter-coder reliability while still allowing us to draw some conclusions about the larger sample as a whole.

The articles were coded independently by two coders (AS and JM for the academic sample, AS and LO for the print media sample), with a third coder (VD) consulted to settle any discrepancies. Our coding structure included the identification of (1) type of source, (2) year of publication, (3) purpose of TMS application, (4) population, (5) overall tone, and (6) specification of TMS parameters. If they were specified in the article, we further coded for what specific parameters were given (i.e., specific forms of TMS, like repetitive TMS (rTMS); frequency of TMS being applied; shape of the TMS coil; and brain region targeted by TMS). The coders then convened to discuss edits that needed to be made to the methods and finalized the coding structure for the larger project sample.

**Fig. 1:**
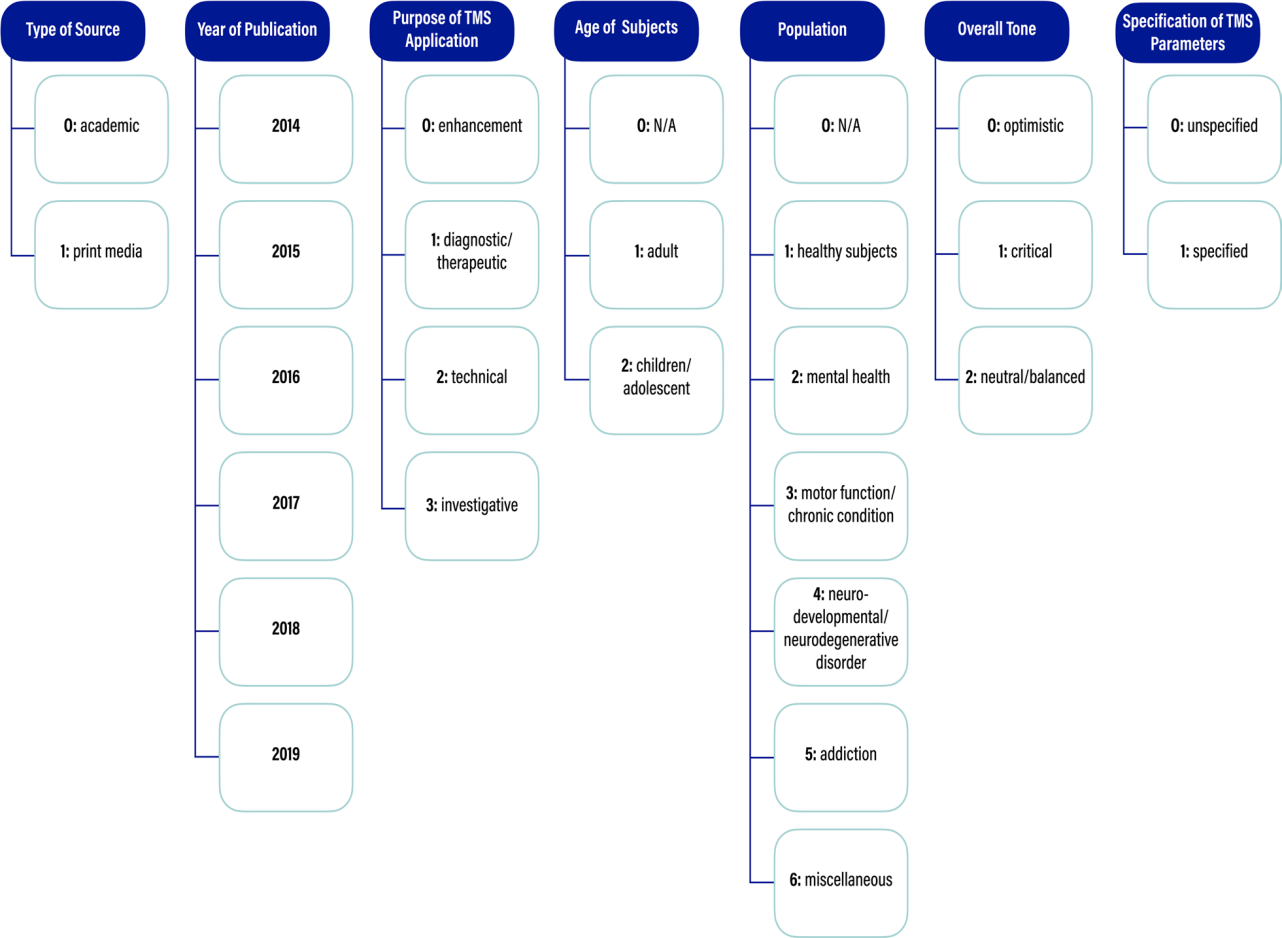
Breakdown of the main codes with quantitative identifiers. Dark blue bubbles represent the seven main codes. Light blue outlined bubbles branching down from the dark blue bubbles represent the subnodes that may be assigned in each main coding category

Ultimately, our coding structure consisted of seven mandatory coding categories for the entire 2100 article sample: (1) type of source, (2) year of publication, (3) purpose of TMS application, (4) age of subjects (5) population, (6) overall tone, and (7) specification of TMS parameters. Figure 1 shows a more extensive breakdown of these code classes and the subnodes that we coded for under each one. These codes were selected because they each pinpointed some point of variability in either the application or the presentation of the technology, best leading us to where the discrepancies in the TMS universe might exist. Namely, since TMS’s reach as a technology is broad, the categories of type of source, year of publication, purpose of TMS application, age of subjects, and population help narrow the specific areas of interest to scientists and the public. Overall tone and specification of TMS parameters allow for examination of the attitudes and specificity the contributing authors take in their writings. Each of the seven codes was marked in every article of the 2100 article sample, and each code was assigned only one subnode per article. Additionally, each of the subnodes was assigned a numerical value^3^ (also shown in Fig. 1 in bold) to allow us to perform additional analyses on our results across the whole sample.

In the subsections below, we provide a brief description of each code and their respective subnodes for the purpose of clarity before presenting our results.

### Type of source

The code for type of source, comprised of the subnodes “academic” or “print media,” corresponds to the broad bodies of literature we sought to evaluate in our search. Articles obtained from PubMed were marked as “academic,” while articles obtained from NexisUni were marked as “print media.” This code was used to divide our sample into the two subsamples to be analyzed.

### Year of publication

This code, comprised of the subnodes “2014,” “2015,” “2016,” “2017,” “2018,” and “2019,” indicates the year that each article was published. The code was intended to illuminate the volume of articles being published each year regarding TMS, which we then take as a quantifiable proxy for measuring popular interest in TMS technology. For consistency in our coding, the academic articles are coded based on the year that they were published in print rather than the year they were published online.

### Purpose of TMS application

The code for purpose of TMS application, comprised of the subnodes “enhancement,” “diagnostic/therapeutic,” “technical,” and “investigative,” was designed to show how TMS is being employed in application (cf. [1]). With it, we sought to understand which applications of TMS are of most interest to both scientists and the general public. Articles coded as “enhancement” indicate that TMS was being used in healthy populations for the purpose of giving people physical, mental, or emotional abilities beyond their natural baseline state (e.g., enhanced memory capabilities). Articles coded as “diagnostic/therapeutic” indicate that TMS was being used either to diagnose or to treat a disease or ailment in non-healthy populations, such as for the treatment of major depressive disorder. Articles coded as “technical” indicate scenarios where TMS was being used or operated to change or better the technology itself. For example, several articles reported on simulated TMS on computer models to compare the effectiveness of different coil shapes in TMS. Finally, articles coded as “investigative” indicate that TMS was being used as a measurement device or probing tool for the sake of understanding different biological processes. Investigative uses include mapping the localization of brain function, understanding disease progressions without the goal of incorporating TMS into treatment, and other such uses.

### Age of subjects

This code, comprised of the subnodes “N/A,” “adult,” and “children/adolescents,” refers to the age range of subjects for whom TMS was being used. The age of subjects helped us determine the general age population that is represented in academic or print media literature on TMS technology in its various applications to help us understand the technology’s current target audience. The subnode “N/A” includes both articles in which no population was used, such as those that demonstrated TMS with a computer model, and review articles, which demonstrated TMS broadly and did not focus on any one particular age range of people. The “adult” subnode refers to articles which either specified a population of 18 years old or older or else centered around a condition that necessitated an older population (e.g., Alzheimer’s disease). The “children/adolescents” subnode, in contrast to this, refers to articles that specified a focus on children, adolescents, or otherwise “young people” in their discussion.

### Population

The population code, comprised of the subnodes “N/A,” “healthy subjects,” “mental health,” “motor function/chronic condition,” “neurodevelopmental/neurodegenerative disorders,” “addiction,” and “miscellaneous,” underscores the broad classes of issues being addressed by TMS. This further narrows the group of people who interact with or benefit from TMS technology in its various applications by illuminating which “categories” the technology served during this period according to the articles in our sample. Articles coded as “N/A” indicate articles that, as stated above, used no population or reviewed several populations. Articles coded as “healthy subjects” indicate those articles in which no underlying condition was being targeted and TMS was being used on healthy individuals. Articles coded as “mental health” indicate that TMS was being used in populations suffering from some form of mental illness or psychiatric condition, such as depression or OCD. Articles given the subnode “motor function/chronic condition” refer to populations suffering from some kind of physical deficit (e.g., stroke) or who are afflicted with an ongoing condition (e.g., fibromyalgia or Parkinson’s disease). Articles coded as “neurodevelopmental/neurodegenerative disorders” indicate TMS was being used to address one of those two types of conditions, including attention-deficit/hyperactivity disorder (ADHD), autism, and dementia or Alzheimer’s disease. Articles coded as “addiction” indicate TMS was being used to address both addictive social behaviors (e.g., gambling) and substance addictions (e.g., smoking, heroin), and finally, the “miscellaneous” subnode covered articles that addressed any condition which did not fit into the prior five codes (e.g., obesity). Articles coded miscellaneous did not receive their own subnode because they occurred in such small numbers (less than 0.5% each) with no discernable link to one another such that their occurrence could not be reported as a trend.

### Overall tone

This code, comprised of “optimistic,” “critical,” and “neutral/balanced” subnodes, contains our assessment of the attitude of each article in our sample. We shifted our focus to what attitude on the whole authors took towards the subject of TMS in order create a top-level understanding of the sentiments of both the print media community and scientific communities. “Optimistic” indicates an overtly positive stance towards TMS technology and refers to articles which either presented only the benefits of TMS and not the drawbacks (e.g., side-effects), or used an abundance of hype language, such as calling TMS a “miracle cure” or “life changing” (see, e.g., [31] in the academic sample and [32] in the print sample). “Critical” indicates an overtly negative stance towards TMS and refers to articles that either overly stressed the drawbacks or dangers of TMS and failed to present the benefits of the technology, or used “doom and gloom” (cf. [21]) language about the technology, such as calling TMS a hoax (see, e.g., [33] in the academic sample and [34] in the print media sample). Finally, neutral/balanced articles adequately presented the benefits of TMS as well as the drawbacks and used no hyperbolic language, contributing to an overall realistic picture of the TMS technology.

### Specification of TMS parameters

Our final code, specification of TMS parameters, was comprised of only two subnodes, “unspecified” and “specified,” and it targeted the degree of specificity authors were using with regards to different TMS paradigms. By ascertaining the degree of specificity, we hoped to understand how well-founded the authors’ conclusions were in scientific backing. As mentioned earlier, we set aside four key pieces of TMS paradigms that we coded where applicable (general TMS paradigm, TMS frequency, shape/orientation of TMS coil, and brain region targeted). Here, articles coded as unspecified indicate that none of the four parameters we outlined were mentioned in the article. In contrast, articles coded as specified mentioned at least one of the parameters.

## Results

In the following subsections, we address our findings for each code, beginning first with the TMS application codes for type of source, year of publication, purpose of TMS application, age of subjects, and population. Then, we address the remaining presentation codes, overall tone and specification of TMS parameters.

### Type of source

The search yielded two samples, 468 print media articles and 1632 academic articles, for a total sample of 2100 articles. Given the large size discrepancy between the two samples, which suggests that the academic community is more interested in TMS than the public, the following results sections provide our data first in the standardized form of percentages so that the two samples could be compared to one another, and secondarily, raw numbers of articles for each subnode are provided.

### Year of publication

**Fig. 2:**
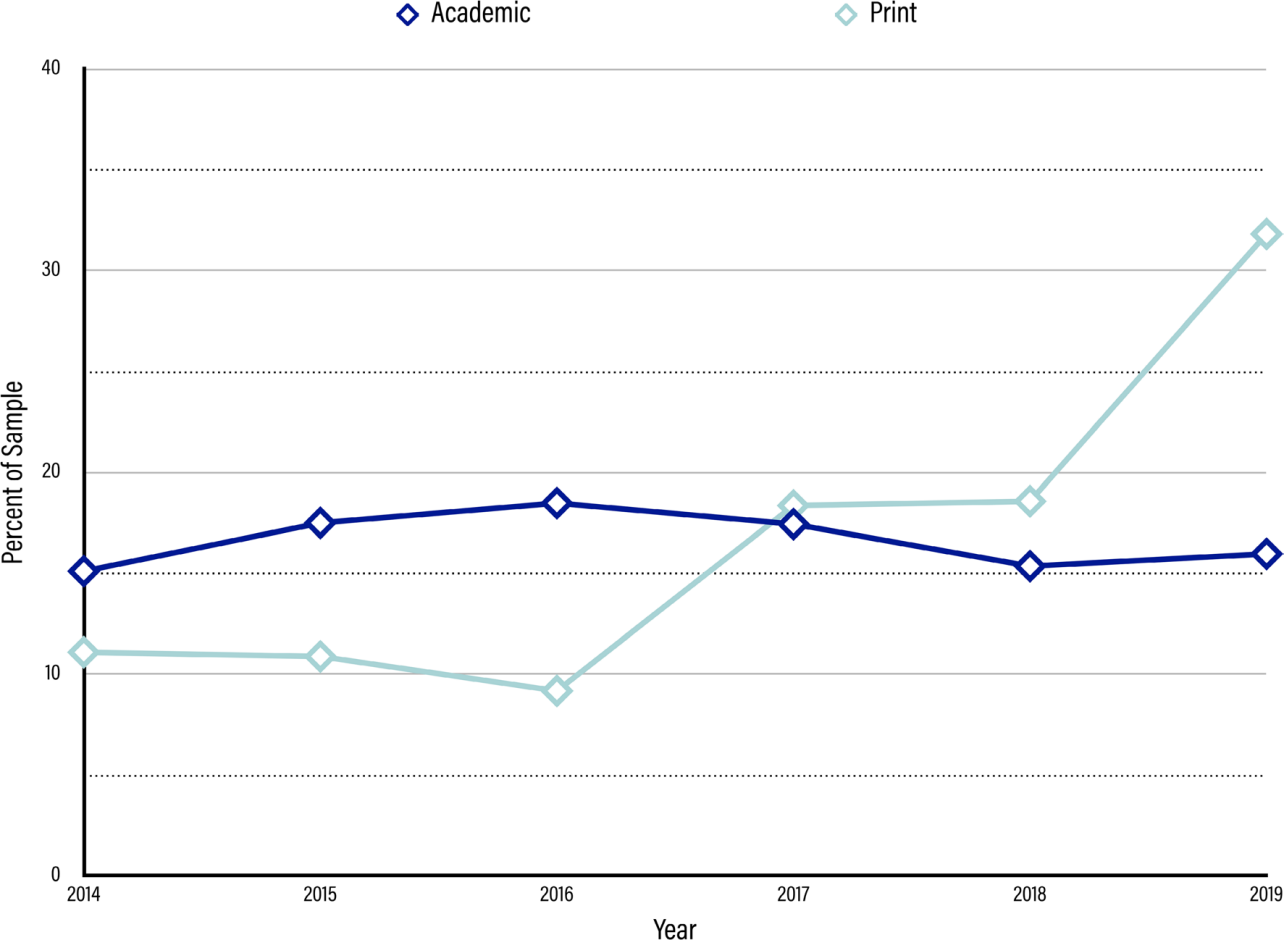
Results for Year of Publication. Dark blue charted line represents the percent of articles per year in the academic media sample. Light blue charted line represents the percent of articles per year in the print media sample

In the academic sample, reporting on TMS trended upward for the first three years (e.g., from 2014 to 2016) before declining again after the nexus in 2016 (shown in Fig. 2). The number of articles reporting on TMS grew from 15.13% (n = 247) of the total sample in 2014 to 17.52% in 2015 (n = 286) before reaching its peak in 2016 at 18.50% (n = 302). After 2016, the trend for the number of articles for each year began a steady decline, shrinking back to 17.46% (n = 285) in 2017 and further to 15.38% (n = 251) in 2018. In 2019, the number of articles rebounded slightly back to 15.99% (n = 261), which might suggest that interest in TMS waxing again.

In the print media sample, we observed a relatively stable amount of reporting in the first three years of our sample followed by a jump in volume (nearly doubling the first three years) in the subsequent three years. The period of 2014 through 2016 exhibited a mild decline, with 2014 representing roughly 11.11% of the print sample (n = 52), 2015 representing 10.90% (n = 51), and 2016 representing 9.19% (n = 43). However, these fluctuations are small enough that we consider these three years to be largely stable. In the following three years, coverage of TMS increased significantly: 2017 and 2018 each yielded roughly 18% of the sample (n = 86, 18.38% and n = 87, 18.59%, respectively), nearly doubling the coverage from 2016. The year 2019 grew even more dramatically, jumping all the way up to 31.84% of our sample (n = 149), which suggests that print media interest in TMS is continuously growing.

### Purpose of TMS application

**Fig. 3:**
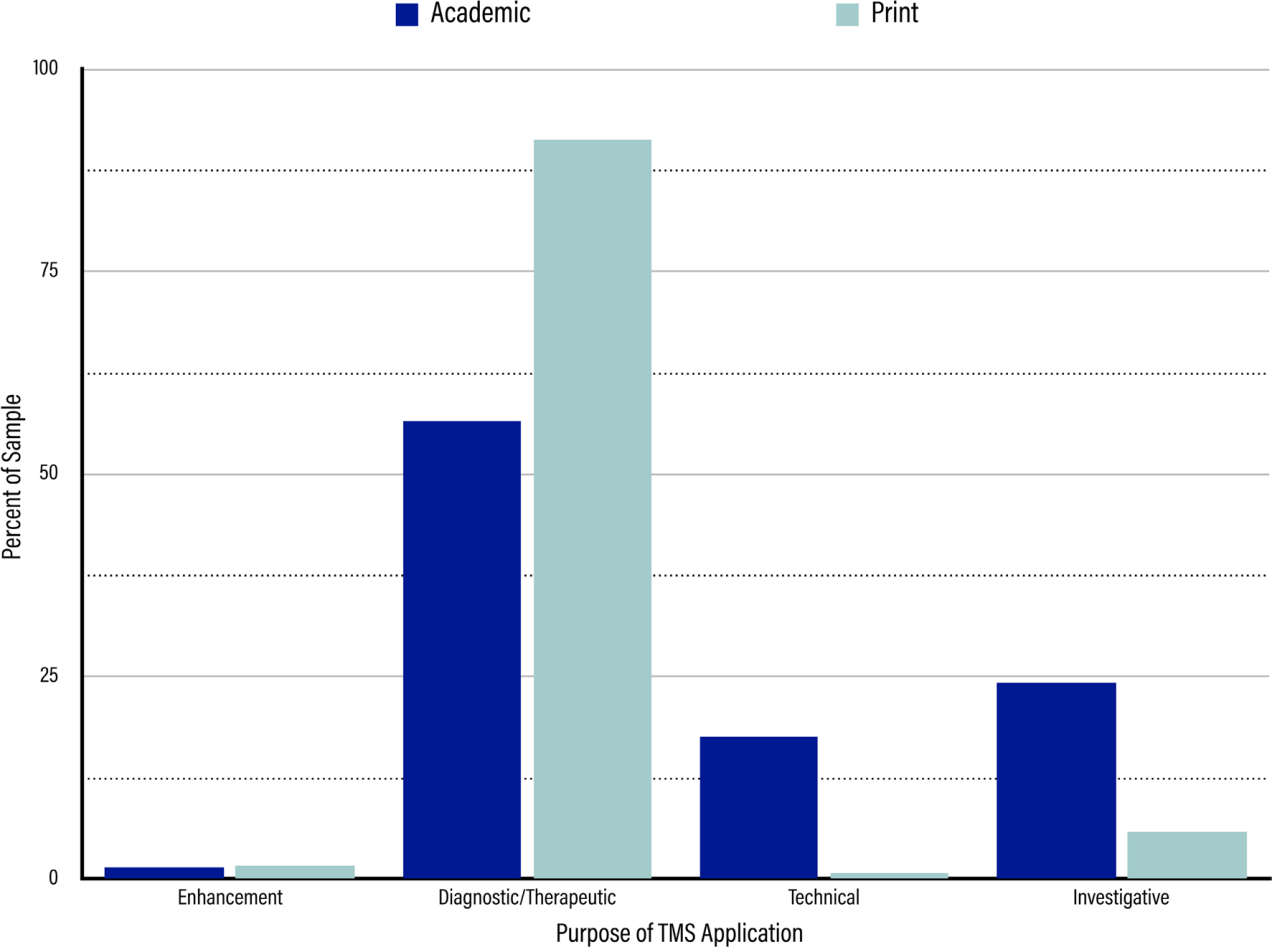
Results for Purpose of TMS Application. Dark blue bars represent the percentage of the sample represented by each purpose in the academic sample. Light blue bars represent the percentage of the sample represented by each purpose in the print media sample. From left to right, the purposes are “Enhancement,” “Diagnostic/Therapeutic,” “Technical,” and “Investigative.”

In the academic sample, the purposes for which TMS was being used were relatively variable (shown in Fig. 3). While a majority of the articles dealt with TMS that was therapeutic in nature (n = 925, 56.68%), the other half of the sample was split somewhat evenly between investigative and technical purposes: 24.27% dealt with TMS for an investigative purpose (n = 396), while 17.53% dealt with a technical purpose (n = 286). As a low-end outlier, 1.53% of our sample dealt with TMS being used for an enhancement purpose (n = 25).

In the print media sample, diagnostic/therapeutic applications of TMS dominated the space. Diagnostic/therapeutic applications made up over 90% of the print sample (n = 428, 91.45%), dwarfing the next largest subnode, investigative uses, which came in at only 5.98% (n = 28). Enhancement and technical applications of TMS represented a negligible portion of the sample, with the enhancement subnode appearing in only 1.71% of the articles (n = 8) and the technical subnode appearing in only 0.86% of the articles (n = 4).

### Age of subjects

In the academic sample, adults comprised the majority of the sample at 78.80% (n = 1286). Children, or participants under the age of 18, comprised only 3.31% of the sample (n = 54). The remaining 17.89% (n = 292) were marked with the subnode N/A because they either were a review article, in which no specific group of participants was mentioned, or dealt with a technical application of TMS that required no participants.

**Fig. 4:**
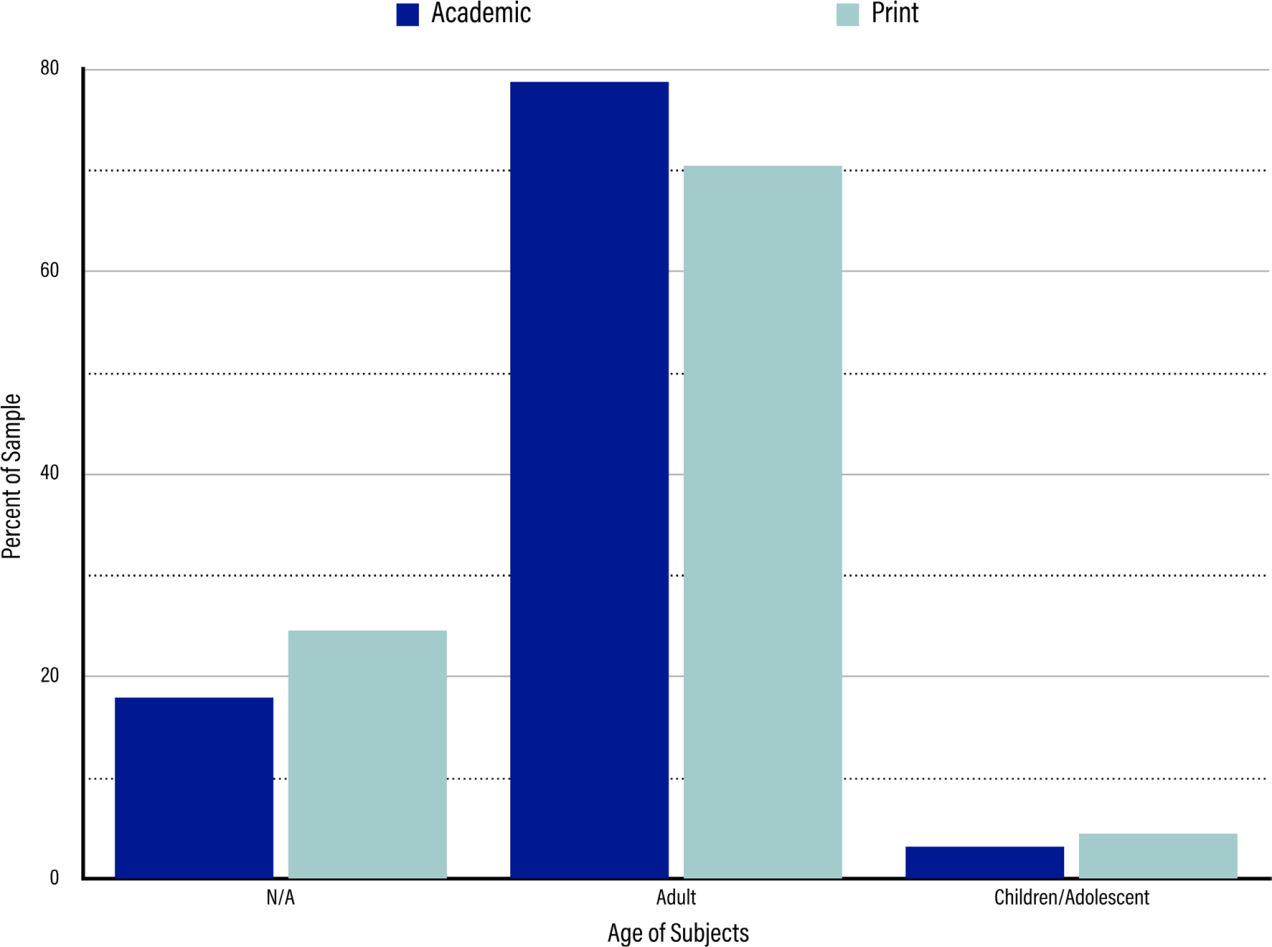
Results for Age of Subjects. Dark blue bars represent the percentage of the sample represented by each age of subjects class in the academic sample. Light blue bars represent the percentage of the sample represented by each age of subjects class in the print media sample. From left to right, the age of subjects classes are “N/A,” “Adult,” and “Child/Adolescent”

In the print media sample, as in the academic sample, adults expectedly accounted for the majority of people for whom the technology was being used, comprising 70.73% of the sample (n = 331). Children and adolescents were the subject of nearly 5% of the articles (n = 21, 4.49%). The rest of the articles in this sample (n = 116, 24.79%) were marked with the subnode N/A because they were either reviews or dealt with a technical application of TMS in which a population was not specified (shown in Fig. 4).

### Population

**Fig. 5:**
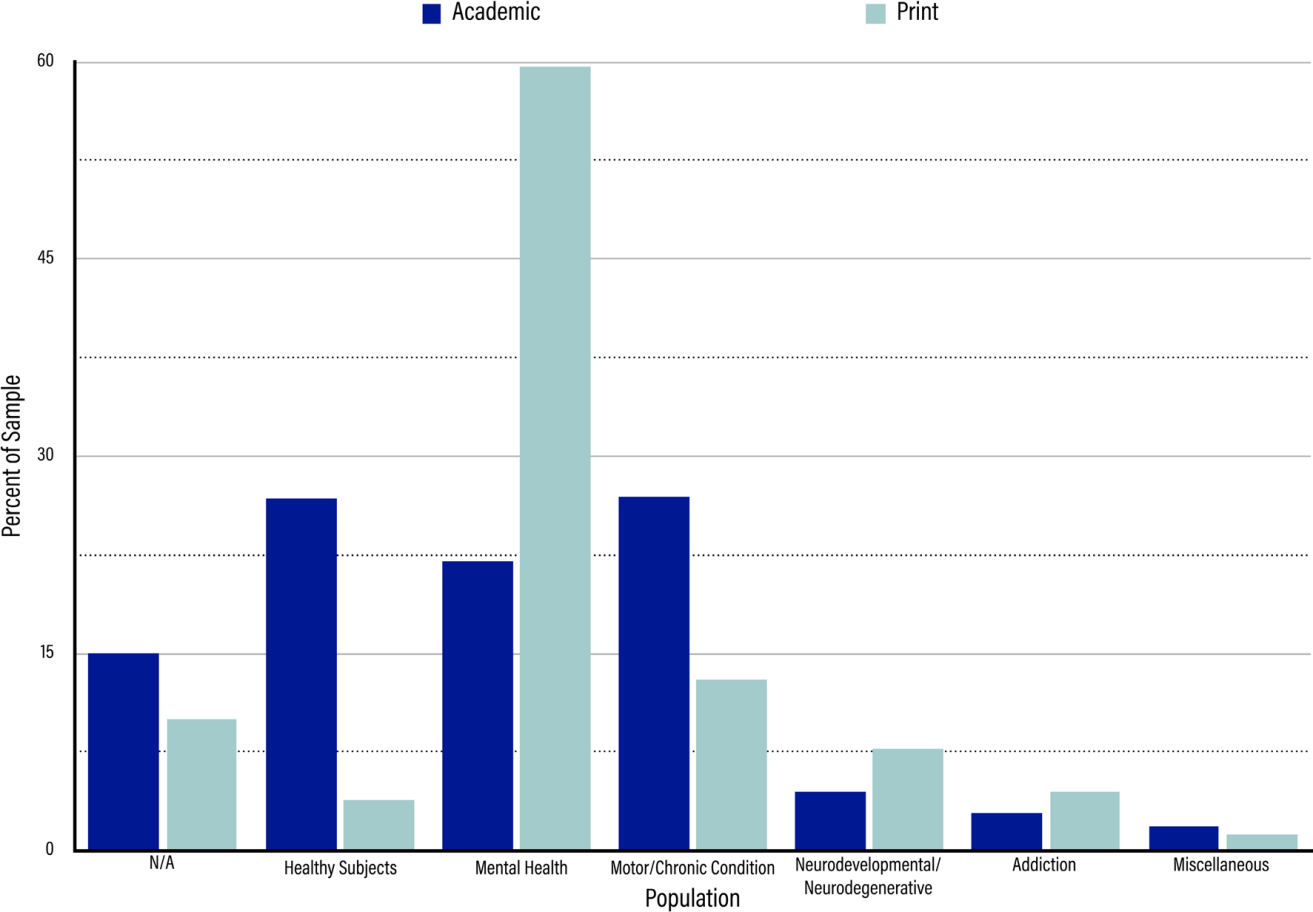
Results for Population. Dark blue bars represent the percentage of the sample represented by each population in the academic sample. Light blue bars represent the percentage of the sample represented by each population in the print media sample. From left to right, the purposes are “N/A,” “Healthy Subjects,” “Mental Health,” “Motor/Chronic Condition,” “Neurodevelopmental/Neurodegenerative,” “Addiction,” and “Miscellaneous.”

In the academic sample, there was considerable variety with regards to the populations TMS was addressing (shown in Fig. 5). Many of the participants noted in the academic articles were healthy participants, which accounted for 26.84% of the population code (n = 438). At around the same level, however, were populations marked as having a motor function or otherwise chronic condition (n = 439, 26.90%) and populations with mental health conditions (n = 359, 22.00%). Significantly lower but still present in the sample were populations marked as neurodegenerative/neurodevelopmental at 4.53% (n = 74), populations dealing with an addiction or substance use/abuse at 2.82% (n = 46), and populations coded as “miscellaneous” at 1.84% (n = 30). Those articles marked as miscellaneous fit into no other delineated subnode but would have accounted for less than 0.5% each if sectioned off into their own subnode. As with age of subjects, the remaining 15.07% (n = 246) were marked as N/A for the same reasons specified above.

**Fig. 6:**
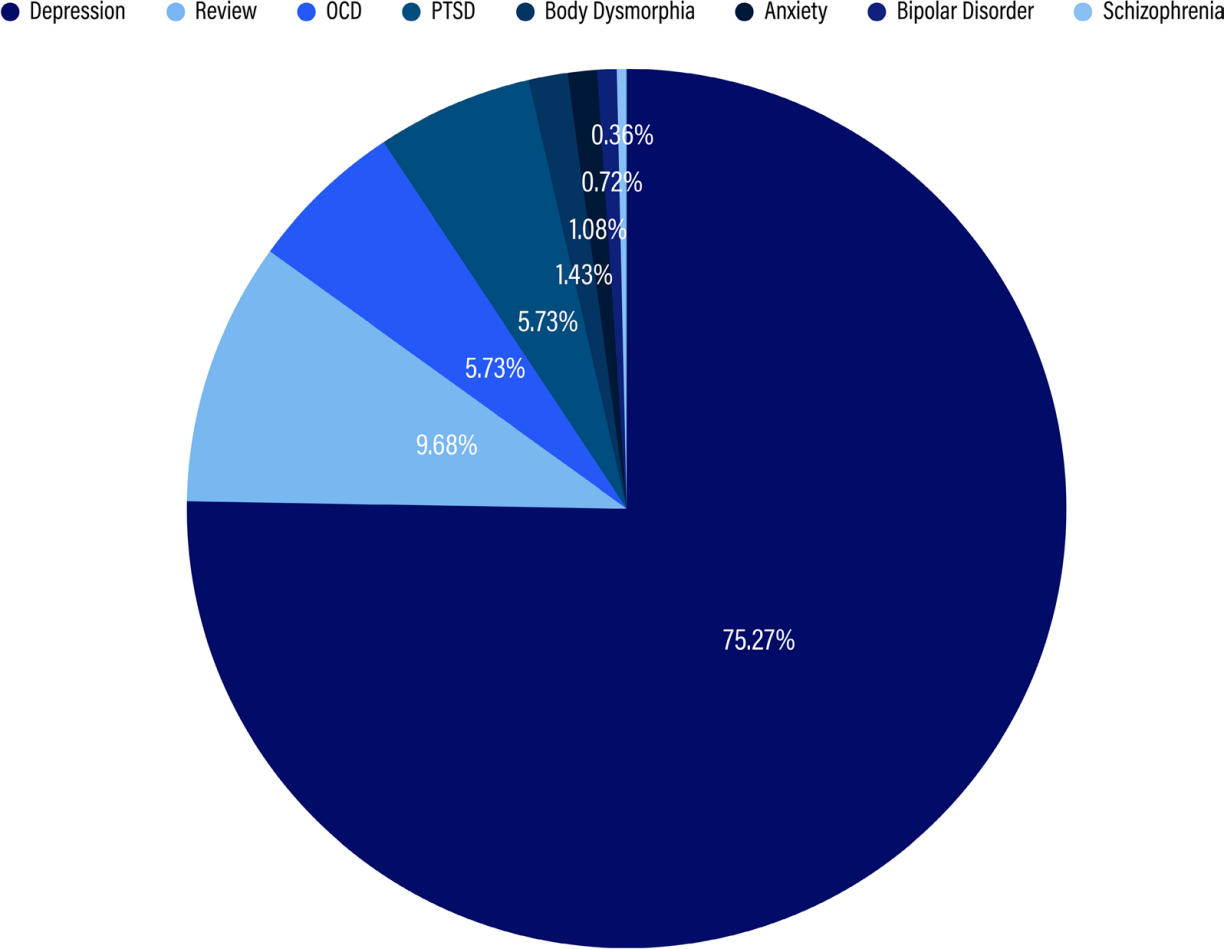
Breakdown of the Mental Health subcode within the print media sample. Each wedge represents what percentage of the Mental Health subcode is occupied by individual mental health conditions. Depression, the largest wedge, represents 75.27%, followed clockwise by Review, 9.68%; OCD, 5.37%; PTSD, 5.37%; Body Dismorphia, 1.43%; Anxiety, 1.08%; Bipolar Disorder, 0.72%; and Schizophrenia, 0.36%

Unlike in the academic sample, the print media sample’s population code was comprised mainly of mental health patients, which tracked with the fact that we found such a strong focus on the therapeutic applications of TMS. Mental health accounted for 59.61% of the sample (n = 279), which was more than four times greater that of any other population subnode. Considering this majority, we broke down the subnode of mental health further into what mental health issues were specifically being targeted with therapies. On the whole, we found that the category of depression accounted for 75.27% of articles in the mental health subnode (n = 210), Obsessive Compulsive Disorder (OCD) accounted for 5.74% of articles (n = 16), Post-Traumatic Stress Disorder (PTSD) accounted for 5.74% of articles (n = 16), body dysmorphia accounted for 1.43% (n = 4), and other mental health issues (e.g., anxiety, bipolar disorder, schizophrenia, etc.) accounted for 2.15% of articles collectively. A further 9.68% (n = 27) of the mental health subnode was made up of reviews, which covered two or more mental health issues. In Fig. 6, shown below, we display how each of these mental health issues was represented in the print media sample.

Aside from mental health, the population subnodes of motor function/chronic conditions and neurodevelopmental/neurodegenerative disorders made up the next two highest categories, with motor function/chronic conditions comprising 13.03% of the articles (n = 60) and neurodevelopmental/neurodegenerative disorders comprising 7.69% (n = 36). The population subnodes of addiction and healthy subjects each accounted for about 4% of the sample (n = 21, 4.49% and n = 18, 3.85%, respectively), while the miscellaneous subnode made up a small 1.28% of the sample (n = 6). As with age of subjects, the remaining 10.04% (n = 47) of the articles were marked with the subnode N/A for the same reasons specified as above.

### Overall Tone

**Fig. 7:**
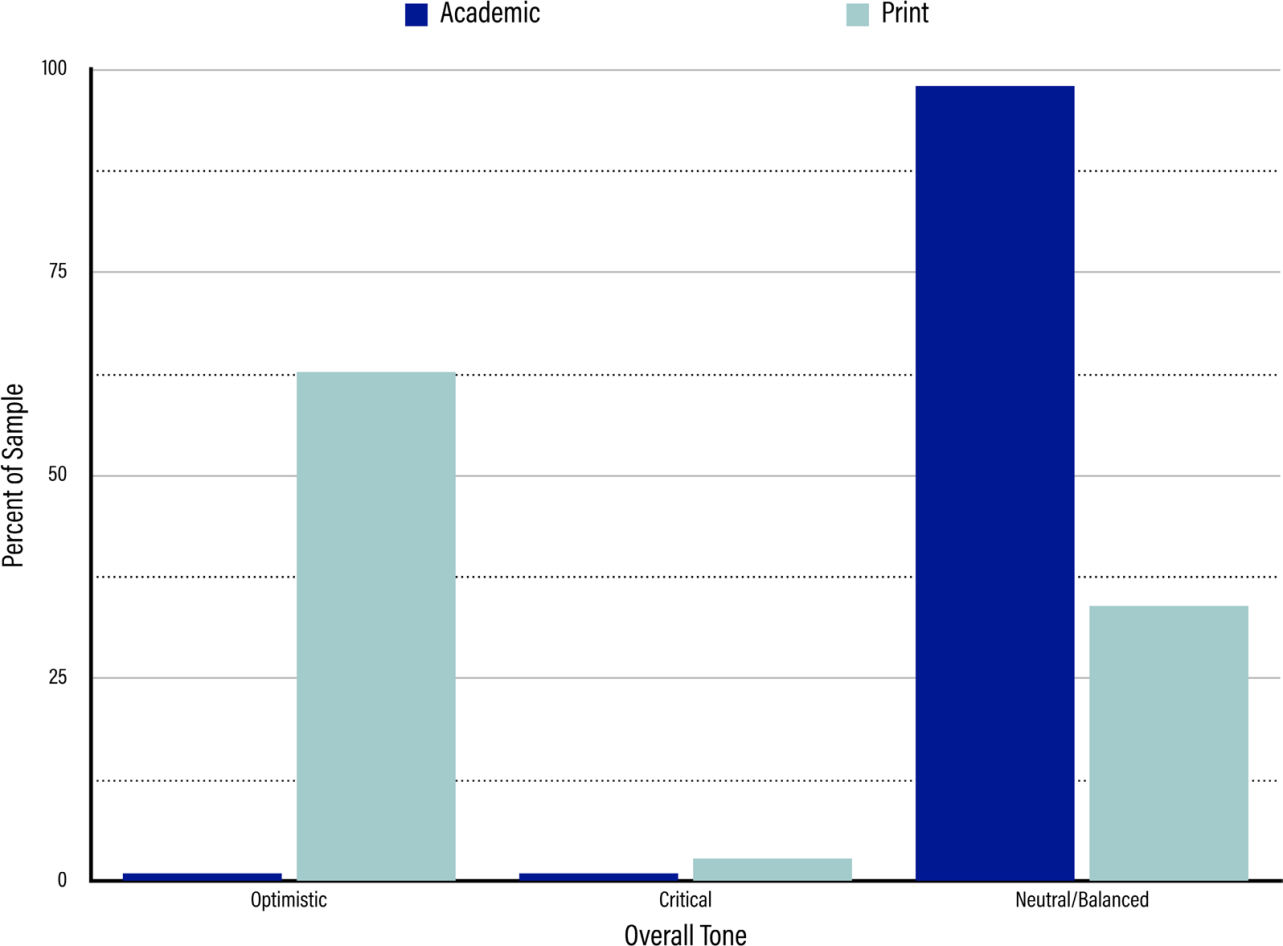
Results for Overall Tone. Dark blue bars represent the percentage of the sample represented by each tone in the academic sample. Light blue bars represent the percentage of the sample represented by each tone in the print media sample. From left to right, the tones are “Optimistic,” “Critical,” and “Neutral/Balanced.”

In the academic sample, as expected, the neutral or balanced tone was dominant (n = 1599, 97.98%). Though some of the articles were coded as either optimistic (n = 17, 1.04%) or critical (n = 16, 0.98%), these were seen largely as outliers in our sample and mostly were linked to review articles that took a stance on TMS one way or the other (shown in Fig. 7).

In the print media sample, on the other hand, optimistic articles far outnumbered the other two subnodes; 63.03% of the articles in the print media sample were optimistic (n = 295), nearly double that of the next closest subnode, neutral/balanced, which accounted for 33.97% of the print media sample (n = 159). In contrast to this, very few articles in the sample took a critical approach to TMS; in total, only 2.99% of the print media sample was marked with the critical subnode (n = 14). Articles in the print media then seemed to give more weight overall to the benefits of TMS than to any concerns surrounding its use.

**Fig. 8:**
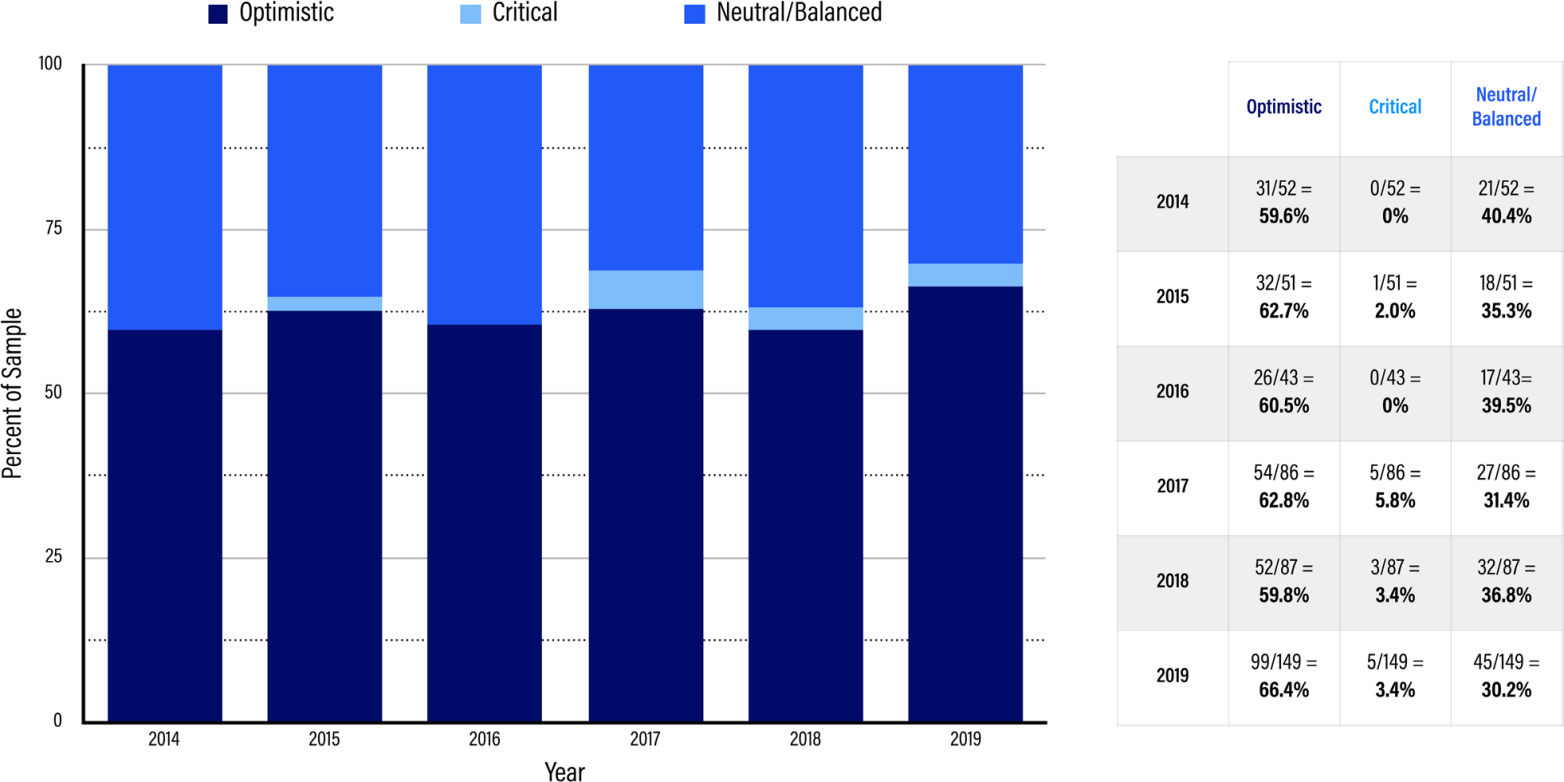
Breakdown of Overall Tone by year within the print media sample. Dark blue bars represent the percentage of articles in the sample in each year which took on a positive tone. The lightest blue bars represent the percentage of articles in the sample in each year which took on a critical tone. The medium blue bars represent the percentage of articles in the sample in each year which took on a neutral or balanced tone. That same data is then represented in a chart to the right

We further investigated tone in the print media sample by breaking it down into tone by year. While each year exhibited a distribution of tone relatively similar to the sample at large, we observed a trend of slight increase in the number of optimistic articles as time went on, which corresponded to a mildly fluctuating decrease in the number of neutral articles over that same period. The breakdown of this sub-analysis can be seen in Fig. 8, shown below.

### Specification of TMS parameters

**Fig. 9:**
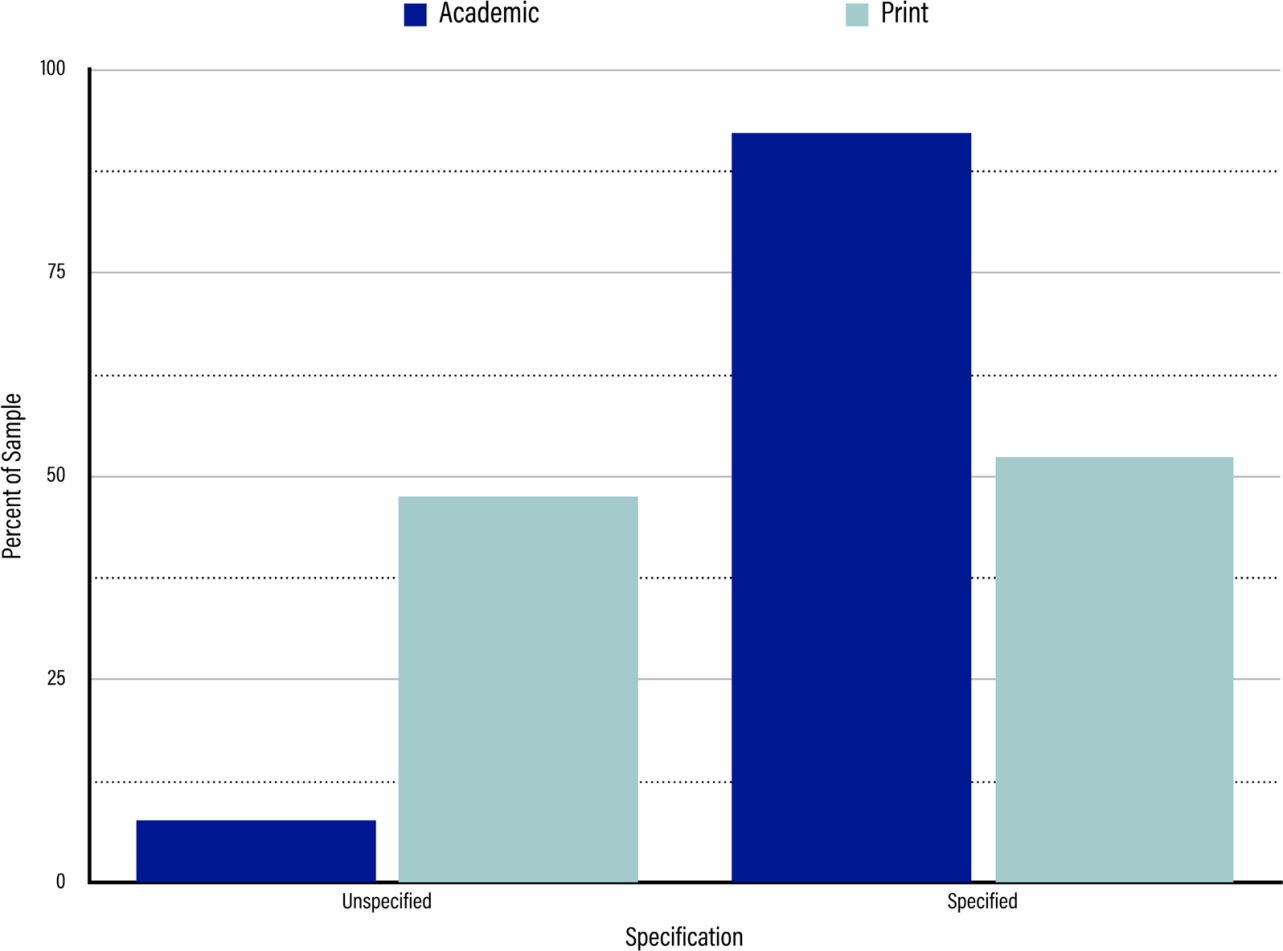
Results for Specification of TMS Parameters. Dark blue bars represent the percentage of the sample represented by each specification status in the academic sample. Light blue bars represent the percentage of the sample represented by each specification status in the print media sample. From left to right, the specification statuses are “Unspecified,” and “Specified.”

In the academic sample, we found the literature to be largely specific in terms of the paradigms and parameters used when applying TMS (shown in Fig. 9). The majority (92.28%, n = 1506) specified at least one, and often more than one, of the four key parameters that we identified. Only 7.72% (n = 126) of the articles did not specify a single TMS parameter, a number consisting mostly of reviews which looked at TMS at large rather than applied any one specific way.

In the print media sample, on the other hand, there was a near even split between articles that failed to specify any TMS parameters and articles that specified at least one of our designated TMS parameters. Nearly half (47.65%) of the print media articles were coded as unspecified and lacked mention of even one TMS parameter (n = 223), and 52.35% of the articles were coded as specified for mentioning at least one (n = 245). Although 245 articles were coded as specified, the vast majority of these articles only specified one of the TMS parameters that we coded for, and none of the articles specified all four of the relevant parameters, signifying that they only minimally met our standards for specification.

## Discussion

Our results indicated several discrepancies between the academic and the print media reporting about TMS technology. With regards to tone, the print media took a staunchly more optimistic stance than the academic literature did. In one sense, this is not surprising, as academic publications which have undergone a peer review process are expected to provide balanced information about benefits and concerns and to include limitations to the study in a tone devoid of hype. While it is the responsibility of the academic press to take a neutral stance, however, the level of optimism from the print media went considerably beyond the level of evidence and confidence concerning benefits of TMS in the academic literature. The print media sample lacked reporting side effects—and, if reported, they were minimized—and offered an abundance of “hype,” with many articles relying on positive user testimonies or anecdotes that extrapolate one person’s positive experience to sell the technology. Concerningly, many of the most optimistic articles about TMS ultimately came from companies and commercial facilities offering TMS treatment^4^; for example, South Bay TMS Therapy Center issued a press release which was published as an article that claimed that “TMS is a very noninvasive procedure that has many benefits to its non-chemical dependent process. It is medication free, FDA approved and proven to be life changing” [35]. Not only does this statement express overt optimism, but it is also patently false; in most cases, TMS does not alleviate the need for medication altogether but is most effective when used as a supplementary treatment. The academic literature has described many limitations of TMS that indicate it is not, in fact, proven to be life changing in the positive sense which is implied here. Most notably, maintenance TMS (or mTMS) is necessary in order to sustain longer term effects of TMS, which means that the long-term side effects of prolonged TMS stimulation [15] —which are largely unknown—are more likely to manifest at a future date.

Our data also showed an increasing trend towards optimism in the print media, with a concomitant decrease in the number of neutral articles, inviting concerns over the accuracy of information being presented to the general public with regards to this technology. This implies that as TMS migrates from being primarily a tool of neuroscience to mainstream clinical practice, the public is more likely to be misinformed by mainstream print media that strays from the evidence available via academic research. Namely, TMS presentations that fail to give a balanced view of the technology leave readers without proper context that would create the guardrails for informed decision making. Reports such as the ones we found in our sample that promise dramatic improvements or overemphasize the probability of dramatic side-effects may overly influence healthcare providers, patients, and family members in weighing the harm-benefit ratio, thereby leading them to disregard stressors (e.g., effects on sleep patterns) that are being introduced by the application of TMS therapy.

Additionally, the academic literature is unsurprisingly more specific about the different TMS paradigms than the print media literature. The print media was characterized by an overall lack of specificity surrounding the details of how TMS ought to be applied, while the academic literature tended to be very specific in its presentation of the TMS paradigms. However, the academic sample did, in fact, exhibit a lack of consensus around how TMS ought to be applied (e.g., in terms of coil placement or frequency of stimulation), even for similar conditions. Paradigms ranged broadly in the academic sample such that no two articles presented the exact same parameters for all four categories, and the strong showing of technical articles still investigating the best way to apply TMS further indicates this lack of agreement. Such a lack of scientific consensus not only raises concerns over how much we actually know about and understand TMS applications, but it also has implications for the wider public. The lack of specificity yet optimistic take on TMS in the print media is backed only by cherry-picked results and opinions from the scientific community, raising further concerns about the legitimacy of the information presented to the public about TMS technology.

Aside from the discrepancies between the two bodies of literature, the academic and print samples exhibited some overall similar trends. In both the academic and print media literature, TMS was being used mainly as a therapeutic tool, but the scope of TMS as a therapy in both was much broader than just the four FDA approved applications. In the academic literature, the populations for whom TMS was being applied ranged across a spectrum of disorders and conditions. This comes to us as rather unsurprising, given that the scientific community is concerned less with rehashing what it knows to be the case already and is concerned more with applying technologies in novel ways and discovering new treatments. Interestingly, though, the variety exhibited in the academic sample played out to a degree in the print media sample, as well. Though TMS as a treatment for depression largely dominated, several other disorders for which TMS is not FDA approved also appeared in a significant amount in the print literature. PTSD, for example, made up 5.74% of the “mental health” code and appeared across the entire 5-year period. Additionally, autism appeared quite frequently in the sample, particularly around the publication of books such as *Switched On: A Memoir of Brain Change and Emotional Awakening* by John Elder Robison [36] covering autism/Asperger’s syndrome and TMS.^5^ These off-label TMS uses raise questions about the ethics of using TMS and reporting on the public use of TMS outside of the FDA-approved applications. Is the safety profile of TMS favorable for uses outside of what is already approved, and what implications does it have for the public if the use of TMS is reported on more broadly?

Finally, a discrepancy in focus of the public and academic debate on the ethics of TMS technology is evident. As a preliminary matter, discussions of the ethics of TMS at all were exceedingly sparse in both bodies of literature, indicating that it is not a priority for either the scientific or media communities at this time. Further, although reviews of TMS and other non-invasive brain stimulation techniques such as transcranial direct current stimulation (tDCS) in the ethics community frequently focus on the enhancement uses, the public discourse on the use of TMS is mostly concerned with therapeutic uses.^6^

While the ethics community at large turns its attention to the morality of creating humans with a “super-brain” and the issues that arise from that, this focus neglects the classic bioethical issues that are perhaps most pertinent to the public currently. Given that some of the earlier neuroscience studies with TMS focused on experiments unlocking savant abilities in healthy people (see e.g., [37, 38]), it is unsurprising that the ethical debate came to focus on enhancement issues. However, it is now clear that the TMS technology is migrating from the realm of exploratory neuroscience into mainstream clinical practice and treatment of psychiatric and neurological conditions. This is evident from the reduction of the number of published studies using TMS from 2016 onward, which is a trend we posit will continue. If this trend continues, important ethical issues may be underexplored, including whether patients know enough to give informed consent to TMS treatment and whether access to TMS therapy is just and beneficent. Additionally, it is unclear what implications reporting of off-label TMS therapy may have for the public at large and what ethical principles bind commercial entities that provide TMS for therapeutic uses. The ethical questions surrounding the use of TMS as a therapeutic technology span much further than the basic question of whether or not TMS is safe to use. As it stands currently, the general public must navigate a less nuanced print media landscape, as well as other sources of public information like direct-to-consumer advertising, of TMS therapy with little guidance from the ethical community.

## Conclusions

If the media are performing an agenda-setting function with TMS treatment, it is presenting an optimistic agenda that exceeds what is supported by the scientific literature and doing so without adequate specification of proper TMS parameters and outside of populations for whom TMS use is currently appropriate. TMS is no longer merely a tool of neuroscience with interesting results in controlled laboratory settings. It is increasingly offered and promoted by commercial entities to the general public as a safe and effective “panacea,” going beyond the FDA-approved treatment modalities. In light of our findings, we suggest a multifaceted approach to handling the social, ethical, and policy issues arising from the evolution of TMS in both public and academic sectors is needed.

In order to facilitate responsible use of these devices, several measures need to be implemented. Increasing neuroscientific literacy for the general public (see [1]) and promoting ethical debate which would inform regulatory bodies monitoring TMS devices and services (see [40, 41]) could help offset negative social outcomes. Also, supporting critical public assessment and engagement [42, 43] as well as responsive and ethical clinical practices might help in balancing the currently unfettered enthusiasm for TMS in the public domain. Specifically, we suggest news reports on health technologies and treatments, like TMS, should include information on the ethical debates that surround these technologies and therapies. Such a call also implicates bioethicists, as we are arguing for both an increased attention to the breadth of ethical issues that surround emerging health technologies and treatments as well as engagement in public discourse on these issues.

Above all, providing adequate information to the public about specific forms of risk associated with TMS is imperative. The risk profile associated with different types of TMS applications varies broadly from relatively high (e.g., seizures due to high intensity rTMS) to relatively low (e.g., single or paired pulse studies in controlled laboratory settings) [44]. As TMS has moved from being solely a research tool of neuroscience into an ever-expanding circle of clinical applications, there are many unknowns that at the same time warrant and impede ethical assessment [45, 46]. These include a lack of knowledge concerning interactions between TMS and other psychiatric medications and treatments [47], possible adverse effects in terms of compromising of tissue in stimulated brain areas [18], and the effects of individual functional anatomy and current oscillatory brain states [48]. There is also a need to increase the understanding of safety and efficacy of treatment with specific parameter variations such as intensity, location, and duration of applied frequencies. Due to this volume of uncertainty and the extent of variability with TMS treatments, it is critical that print media and other popular news sources be given accurate, comprehensive, and transparent information when engaging with the subject of TMS.

Ultimately, presenting TMS to public audiences as simply a “safe and effective” treatment belies the scientific complexity and is arguably unethical. We call upon the ethics community to increase scrutiny of TMS services in order to ensure that peoples’ knowledge of health technologies is not unduly influenced by sensational claims and a general lack of adequate information.

## Data Availability

The datasets used and/or analyzed during the current study are available from the corresponding author on reasonable request.

## Abbreviations

TMS: Transcranial magnetic stimulation
mTMS: Maintenance TMS
rTMS: Repetitive TMS
FDA: (United States) Food and Drug Administration
MD: Major depression
OCD: Obsessive–compulsive disorder
PTSD: Post-traumatic stress disorder
AST: Agenda-setting theory
tDCS: Transcranial direct current stimulation
ADHD: Attention-deficit/hyperactivity disorder

## Footnotes

1 The Clinical TMS Society, a trade association, assists its members in optimizing the administration of TMS, developing novel indications for TMS therapy, expanding insurance coverage, increasing public awareness of TMS, and advertising individual TMS clinics. See https://www.clinicaltmssociety.org/about

2 Searched: ( "Transcranial Magnetic Stimulation/adverse effects"[Mesh] OR "Transcranial Magnetic Stimulation/classification"[Mesh] OR "Transcranial Magnetic Stimulation/drug effects"[Mesh] OR "Transcranial Magnetic Stimulation/economics"[Mesh] OR "Transcranial Magnetic Stimulation/ethics"[Mesh] OR "Transcranial Magnetic Stimulation/history"[Mesh] OR "Transcranial Magnetic Stimulation/instrumentation"[Mesh] OR "Transcranial Magnetic Stimulation/methods"[Mesh] OR "Transcranial Magnetic Stimulation/nursing"[Mesh] OR "Transcranial Magnetic Stimulation/organization and administration"[Mesh] OR "Transcranial Magnetic Stimulation/pharmacology"[Mesh] OR "Transcranial Magnetic Stimulation/psychology"[Mesh] OR "Transcranial Magnetic Stimulation/radiation effects"[Mesh] OR "Transcranial Magnetic Stimulation/standards"[Mesh] OR "Transcranial Magnetic Stimulation/statistics and numerical data"[Mesh] OR "Transcranial Magnetic Stimulation/therapeutic use"[Mesh] OR "Transcranial Magnetic Stimulation/therapy"[Mesh] OR "Transcranial Magnetic Stimulation/trends"[Mesh]).

3 Year of publication was not assigned a separate numerical value since the years themselves served as their own numerical code.

4 It should be noted that distinguishing between promotional material from companies and journalism is particularly important. Unfortunately, this is not straightforward, as some journalists take over company (and university) issued press releases word-for-word. Therefore, more research is needed to (potentially) rank journalistic sources on originality in the future. We thank an anonymous reviewer for constructive comments that prompted us to make this clear.

5 Some of these articles are about the book and the contents of it, and some use the timing of the book’s publication to talk about this topic. Other articles talk about autism and TMS with no relation to the book.

6 Ethics literature on TMS for therapeutic purposes—including off label uses—has grown in recent years (see, e.g., [39]), though it has yet to catch up to the landscape of quandaries the academic and popular media literature have set forth.
